# Marker-trait association analysis for drought tolerance in smooth bromegrass

**DOI:** 10.1186/s12870-021-02891-0

**Published:** 2021-02-25

**Authors:** F. Saeidnia, M. M. Majidi, A. Mirlohi

**Affiliations:** Department of Agronomy and Plant Breeding, College of Agriculture, Isfahan University of Technology, Isfahan, 84156-83111 Iran

**Keywords:** Association analysis, Population structure, Sequence-related amplified polymorphism, Smooth bromegrass, Water deficit

## Abstract

**Background:**

Little information is available on the application of marker-trait association (MTA) analysis for traits related to drought tolerance in smooth bromegrass. The objectives of this study were to identify marker loci associated with important agronomic traits and drought tolerance indices as well as fining stable associations in a diverse panel of polycross derived genotypes of smooth bromegrass. Phenotypic evaluations were performed at two irrigation regimes (normal and deficit irrigation) during 2 years; and association analysis was done with 626 SRAP markers.

**Results:**

The results of population structure analysis identified three main subpopulations possessing significant genetic differences. Under normal irrigation, 68 and 57 marker-trait associations were identified using general linear model (GLM) and mixed linear mode1 (MLM), respectively. While under deficit irrigation, 61 and 54 markers were associated with the genes controlling the studied traits, based on these two models, respectively. Some of the markers were associated with more than one trait. It was revealed that markers Me1/Em5–11, Me1/Em3–15, and Me5/Em4–7 were consistently linked with drought-tolerance indices.

**Conclusion:**

Following marker validation, the MTAs reported in this panel could be useful tools to initiate marker-assisted selection (MAS) and targeted trait introgression of smooth bromegrass under normal and deficit irrigation regimes, and possibly fine mapping and cloning of the underlying genes and QTLs.

**Supplementary Information:**

The online version contains supplementary material available at 10.1186/s12870-021-02891-0.

## Background

Smooth bromegrass (*Bromus inermis* Leyss.) is a long-lived and cool-season grass species [1, 2] which is adapted to dry regions and grown mostly for hay, pasture and soil conservation [1].

Drought is the major abiotic constraint affecting growth and productivity of crops worldwide [3, 4]. Development of suitable cultivars with more drought tolerance is crucial for enhancing sustainable production of plants and provides a strategy for alleviation of the effects of climate change [5]. However, drought tolerance is a complex and quantitative trait, including several metabolic pathways. A promising strategy to facilitate selection for drought tolerance is to identify and select for genetic markers linked to the traits related to drought tolerance (marker assisted selection; MAS). The main prerequisite for MAS is the availability of markers that are tightly linked to genes or quantitative trait loci (QTLs) which can be used to select for traits that are difficult to measure or dependent on the developmental stage [6, 7].

The application of molecular markers allows locating the genes of interest in the genome, thus avoiding the time and the space needed in breeding programs [8]. Sequence-related amplified polymorphism (SRAP) is a PCR-based molecular marker technique which can be used for a variety of purposes, including genetic diversity analysis, map construction, gene tagging, genomic and cDNA fingerprinting, and map based cloning [9, 10]. Moreover, it is an advanced molecular marker for genetic research in grass and forage species, which uses from two primers with an arbitrary nucleotide sequence and therefore can detect nucleotide sequence polymorphisms [11]. Analysis of SRAP data has frequently been used for the construction of linkage maps [12, 13] and identification of QTLs [14, 15]. However, the utilization of SRAP for grass and forage researches such as association analysis in smooth bromegrass is rare.

The first step towards MAS, as an important tool for accelerating varietal improvement and rate of genetic gain, is to dissect marker-trait associations (MTAs) [16]. Two approaches which can be used for dissection of quantitative complex traits are association mapping and linkage analysis [17, 18]. However, QTL mapping has a low resolution and requires a lot of time and resources [19, 20]. Genome-wide association studies (GWAS) or association mapping have recently become a popular alternative to QTL mapping for identifying MTAs in plant populations [21, 22]. Compared to linkage mapping, GWAS overcomes several of the drawbacks of QTL mapping. It offers higher mapping resolution, is less time consuming and requires fewer resources, and evaluates a wide range of alleles rapidly [23, 24].

In order to avoid identifying spurious associations between markers and traits and also to avoid of both types of error (types I and II) in association analysis, it is necessary to evaluate the population structure and use from a mixed-model approach [6, 25, 26]. Two models of general linear model (GLM) and mixed linear model (MLM) are used for association analysis. In MLM, both the kinship matrix (K) and population structure (Q) are merged, whereas in the GLM, only population structure information is utilized as a covariate [6].

In recent years, the application of association analysis in forage grasses is discussed in several reports. In perennial ryegrass (*Lolium perenne*) the application of association mapping for some traits such as flowering time, leaf length, carbohydrate content, submergence tolerance, salinity and drought tolerance has been evaluated [27–31]. In tall fescue (*Festuca arundinacea*), SSR loci related to agronomic traits [32] and heat-tolerance-related traits [33] have been detected. Kempf et al. (2017) applied SRAP and SSR markers in marker–trait association analysis for agronomic and compositional traits of sainfoin (*Onobrychis viciifolia*) [7]. In orchardgrass, studies have been carried out for detection of the loci related to drought tolerance, seed yield, forage yield [34], rust resistance [35], and heading date [36]. Such studies have demonstrated that GWAS is an efficient method for identifying genomic regions associated with complex quantitative traits. However, in smooth bromegrass the application of association mapping in identifying links between genes or markers with complex quantitative traits such as drought tolerance is still in its infancy. This study was conducted to: i) identify genetic loci associated with the key agronomic traits and drought tolerance, under normal and water deficit conditions using SRAP markers; and ii) discover stable marker loci linked to the agronomic and drought tolerance related traits.

## Results

### Phenotyping

Significant differences were observed between irrigation regimes for most of the measured traits. Except for flag leaf length (FLL), flag leaf width (FLW), panicle length (PL), and winter growth vigor (WGV), the magnitude of mean performance was significantly decreased for all of the evaluated traits, under water-deficit condition. Dry matter yield (DMY) was decreased by deficit irrigation 42% on average (Table 1).

**Table 1:** Mean performance, phenotypic coefficient of variation (PCV), genotypic coefficient of variation (GCV), and broad- sense heritability (h^2^_b_) of traits recorded under normal and water deficit conditions in smooth bromegrass genotypes

Traits	Mean ± SD		Change (%)	PCV (%)		GCV (%)		h^2^_b_ (%)		
	Normal	Stress		Normal	Stress	Normal	Stress	Combined	Normal	Stress
DPE (day)	58.89 ± 7.62	56.61 ± 6.84	3.87^a^	9.37	10.49	8.95	10.24	95.32	91.27	95.13
DA (day)	80.36 ± 6.83	77.83 ± 6.17	3.15^a^	6.55	7.07	6.35	6.89	95.60	94.05	95.06
PH (cm)	96.57 ± 16.22	75.55 ± 13.69	21.77^b^	6.08	12.41	4.89	11.68	73.05	64.72	88.59
FLL (mm)	141.62 ± 28.40	152.54 ± 29.96	−7.71^a^	10.36	11.50	8.88	10.26	84.73	73.46	79.62
FLW (mm)	6.99 ± 1.33	7.18 ± 1.73	−2.72^n.s^	12.25	14.39	11.76	13.46	92.73	92.17	87.51
PL (cm)	16.03 ± 2.13	16.00 ± 2.34	0.19^n.s^	9.14	10.21	8.42	9.42	89.08	84.92	85.17
NS (No. plant^−1^)	187.58 ± 78.75	149.57 ± 68.14	20.26^b^	27.32	30.87	23.77	27.42	86.40	75.75	78.89
DMY (g/plant)	99.25 ± 46.61	57.12 ± 28.75	42.45^b^	23.13	27.45	19.09	22.30	77.99	68.13	66.01
CD (cm)	25.76 ± 5.25	23.28 ± 4.19	9.63^b^	14.54	12.97	13.74	12.02	89.66	89.34	85.89
WGV	2.47 ± 0.97	3.58 ± 1.35	−44.94^b^	20.66	19.88	16.18	16.84	75.19	61.33	71.80

*GCV* Genotypic coefficient of variation, *PCV* Phenotypic coefficient of variation, *SD* Standard deviation

*CD* Crown diameter, *DA* Days to anthesis, *DMY* Dry matter yield, *DPE* Days to panicle emergence, *FLL* Flag leaf length, *FLW* Flag leaf width, *NS* Number of stems per plant, *PH* Plant height, *PL* Panicle length, *WGV* Winter growth vigor

^a^, ^b^, and ^c^ significant at the 0.05, 0.01, and 0.001 probability levels, respectively; ns: not significant

Phenotypic coefficient of variation (PCV) showed a range from 4.89% for plant height (PH) to 23.77% for number of stems per plant (NS) under normal irrigation and from 6.89% for days to anthesis (DA) to 27.42% for NS under deficit irrigation (Table 1). Genetic coefficient of variation (GCV) ranged from 6.08% for PH to 27.32% for NS under normal irrigation and from 7.07% for DA to 30.87% for NS under deficit irrigation. Except for crown diameter (CD), the values of genetic variation under deficit irrigation were higher than the ones for normal irrigation (Table 1).

The estimates of broad-sense heritability for each irrigation regime are given in Table 1. Moderate to high values of heritability estimates were observed for all of the evaluated traits, under both irrigation regimes. The range of this parameter was from 61.33% for WGV to 94.05% for DA under normal irrigation and from 66.01% for DMY to 95.13% for days to panicle emergence (DPE) under deficit irrigation regime. For all traits, estimates of heritability were higher under normal irrigation than deficit irrigation (Table 1).

### Genotyping

In total, 959 bands were created from 30 SRAP primer combinations, of which 626 bands were polymorphic (Table 2). The range of total number of bands scored per primer combination was from 13 (Me3/Em3) to 28 (Me4/Em2), with an average of 21 bands. The percentage of polymorphic bands ranged from 55.17% (Me4/Em3) to 85.19% (Me3/Em4) with an average of 69.85%. The relative informativeness of each marker can be evaluated based on its PIC value. In the present study, PIC value ranged from 0.35 (Me1/Em5) to 0.50 (Me2/Em4, Me3/Em4, and Me5/Em3), with the average of 0.45. The highest and lowest MI values were 12.60 (Me4/Em2) and 5.85 (Me3/Em3), respectively. Markers Me4/Em2 and Me3/Em1 showed the highest and lowest R*P* values, respectively (Table 2).

**Table 2:** Information and diversity statistics for sequence related amplified polymorphism (SRAP) markers used for association analysis in smooth bromegrass

No.	Oligo name	Oligo sequence 5′ → 3′	NPB/NB	PPB	PIC	MI	RP
1	Me1/Em1	TGAGTCCAAACCGGTTG GACTGCGTACGAATTTGC	24/33	72.73	0.47	11.28	23.94
2	Me1/Em2	TGAGTCCAAACCGGTTG GACTGCGTACGAATTACG	26/36	72.22	0.43	11.18	24.61
3	Me1/Em3	TGAGTCCAAACCGGTTG GACTGCGTACGAATTTAG	23/32	71.88	0.49	11.27	20.06
4	Me1/Em4	TGAGTCCAAACCGGTTG GACTGCGTACGAATTCAG	19/27	70.37	0.44	8.36	19.78
5	Me1/Em5	TGAGTCCAAACCGGTTG GACTGCGTACGAATTCGA	24/37	64.86	0.35	8.40	25.83
6	Me1/Em6	TGAGTCCAAACCGGTTG GACTGCGTACGAATTTGA	25/37	67.57	0.37	9.25	26.72
7	Me2/Em1	TGAGTCCAAACCGGTGT GACTGCGTACGAATTTGC	20/31	64.52	0.49	9.80	23.00
8	Me2/Em2	TGAGTCCAAACCGGTGT GACTGCGTACGAATTACG	19/27	70.37	0.49	9.31	22.22
9	Me2/Em3	TGAGTCCAAACCGGTGT GACTGCGTACGAATTTAG	22/31	70.97	0.38	8.36	21.50
10	Me2/Em4	TGAGTCCAAACCGGTGT GACTGCGTACGAATTCAG	26/34	76.47	0.46	11.96	27.50
11	Me2/Em5	TGAGTCCAAACCGGTGT GACTGCGTACGAATTCGA	24/37	64.86	0.48	11.52	29.17
12	Me2/Em6	TGAGTCCAAACCGGTGT GACTGCGTACGAATTTGA	18/25	72.00	0.50	9.00	17.00
13	Me3/Em1	TGAGTCCAAACCGGATA GACTGCGTACGAATTTGC	14/19	73.68	0.46	6.44	12.50
14	Me3/Em2	TGAGTCCAAACCGGATA GACTGCGTACGAATTACG	18/22	81.82	0.49	8.82	22.80
15	Me3/Em3	TGAGTCCAAACCGGATA GACTGCGTACGAATTTAG	13/16	81.25	0.45	5.85	16.70
16	Me3/Em4	TGAGTCCAAACCGGATA GACTGCGTACGAATTCAG	23/27	85.19	0.50	11.50	26.90
17	Me3/Em5	TGAGTCCAAACCGGATA GACTGCGTACGAATTCGA	16/21	76.19	0.46	7.36	19.70
18	Me3/Em6	TGAGTCCAAACCGGATA GACTGCGTACGAATTTGA	15/19	78.95	0.41	6.15	18.60
19	Me4/Em1	TGAGTCCAAACCGGAGC GACTGCGTACGAATTTGC	19/30	63.33	0.44	8.36	20.00
20	Me4/Em2	TGAGTCCAAACCGGAGC GACTGCGTACGAATTACG	28/45	62.22	0.45	12.60	31.61
21	Me4/Em3	TGAGTCCAAACCGGAGC GACTGCGTACGAATTTAG	16/29	55.17	0.45	7.20	18.17
22	Me4/Em4	TGAGTCCAAACCGGAGC GACTGCGTACGAATTCAG	23/34	67.65	0.43	9.89	26.00
23	Me4/Em5	TGAGTCCAAACCGGAGC GACTGCGTACGAATTCGA	20/29	68.97	0.39	7.80	20.94
24	Me4/Em6	TGAGTCCAAACCGGAGC GACTGCGTACGAATTTGA	23/39	58.97	0.47	10.81	28.22
25	Me5/Em1	TGAGTCCAAACCGGTGC GACTGCGTACGAATTTGC	25/37	67.57	0.46	11.50	26.44
26	Me5/Em2	TGAGTCCAAACCGGTGC GACTGCGTACGAATTACG	21/30	70.00	0.47	9.87	15.78
27	Me5/Em3	TGAGTCCAAACCGGTGC GACTGCGTACGAATTTAG	23/33	69.70	0.50	11.50	22.33
28	Me5/Em4	TGAGTCCAAACCGGTGC GACTGCGTACGAATTCAG	24/33	72.73	0.40	9.60	23.50
29	Me5/Em5	TGAGTCCAAACCGGTGC GACTGCGTACGAATTCGA	19/32	59.38	0.49	9.31	21.83
30	Me5/Em6	TGAGTCCAAACCGGTGC GACTGCGTACGAATTTGA	16/25	64.00	0.48	7.68	13.06

*MI* Marker index, *NB* No. of bands, *NPB* No. of polymorphic bands, *PIC* Polymorphic information content, *PPB* Percentage of polymorphic bands, *RP* Resolving power

### Population structure and association analysis

**Fig. 1:**
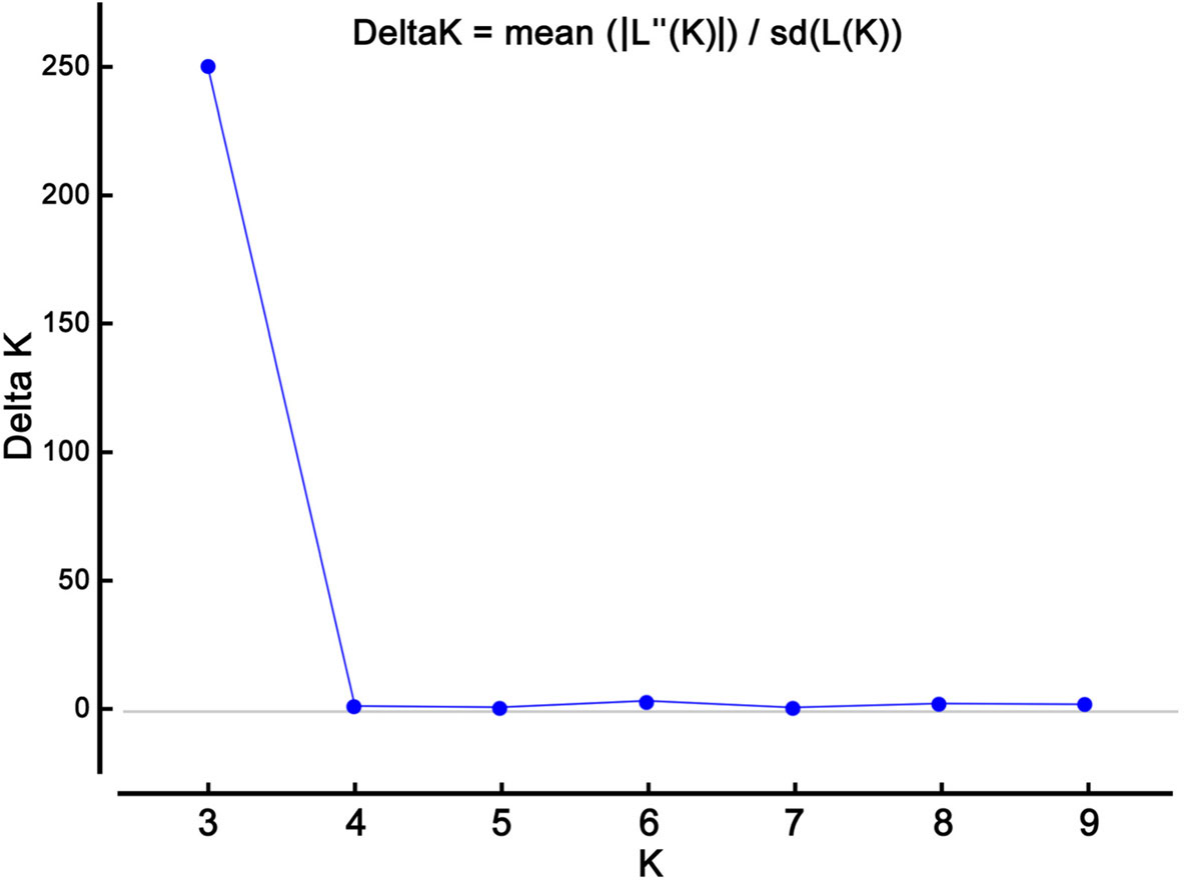
Population structure analysis in a diverse germplasm of smooth bromegrass (The optimum number of subpopulations was determined using Δk in the Bayesian clustering method)

**Fig. 2:**
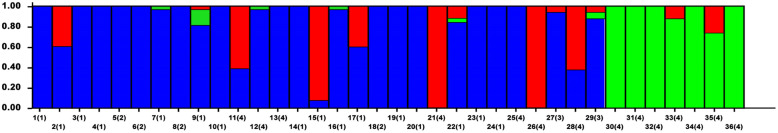
The results of genetic association analysis of smooth bromegrass genotypes performed by STRUCTURE software version 2.3.4. The membership coefficients of genotypes are given on the *y*-axis. Each of the three identified subpopulations is shown by a different color

The maximum likelihood and D*K* were used to calculate the optimum number of subpopulations (*K*). The maximum value of D*K* obtained at *K* = 3, suggested that there were three subpopulations in the smooth bromegrass panel (Table 3; Figs. 1 and 2). Each of these three subpopulations had its own characteristics. Subpopulation 1 contained seven genotypes of G30, G31, G32, G33, G34, G35, and G36 (Fig. 2). All of these genotypes were belonged to Hungary, and had higher persistence than other genotypes. Subpopulation 2 included genotypes G11, G15, G21, G26, and G28, which all of them were belonged to Hungary except for G15 (Iranian genotype). Genotypes of this subpopulation were early flowering and had lower productivity than other genotypes. The remaining genotypes were located in the third subpopulation (Fig. 2). This subpopulation showed late flowering and had higher productivity than other ones. All of the genotypes of this subpopulation were Iranian except for G12, G13, and G25 (Hungarian genotypes). As observed, structure analysis was able to separate genotypes based on geographical or ecological data.

**Table 3:** Calculated statistics to detect optimum number of subpopulations (*K*) in structure analysis of smooth bromegrass genotypes using the STRUCTURE program

K	Reps.	Mean LnP (K)	Stdev. LnP (K)	Ln^′^ (K)	Ln^′′^ (K)	ΔK
2	5	−15,785.10	8.81	–	–	–
3*	5	−15,648.70	18.45	136.40	4616.68	250.19
4	5	−20,128.98	5968.36	− 4480.28	5961.88	1.00
5	5	−18,647.38	4062.75	1481.60	1102.94	0.27
6	5	−18,268.72	2143.97	378.66	5471.46	2.55
7	5	−23,361.52	5990.88	− 5092.80	3066.18	0.51
8	5	−25,388.14	5288.39	− 2026.62	8905.46	1.68
9	5	−36,320.22	8671.94	−10,932.08	15,386.38	1.77
10	5	−31,865.92	4503.99	4454.30	–	–

Mean LnP (*K*), mean of LnP(D) of repetitions for each *K*; Stdev. LnP (*K*), standard deviation of repetitions; Ln′ (*K*), Ln (*K*) *n* – Ln (*K*) *n* – 1; Ln′′ (*K*), Ln′ (*K*) *n* – Ln′ (*K*) *n* – 1; D*K*, |Ln′′ (*K*)|/stdev LnP (*K*). *, *K*-value with largest D*K*

Association analysis between SRAP markers and the phenotypic mean of traits (over years and irrigation regimes) was separately conducted based on both GLM and MLM models. Under normal irrigation, based on the GLM model (*P* values < 0.01 and a cut-off value of 0.05 for the FDR) 68 SRAP markers showed significant associations with means of the studied traits, at the 0.01 probability level (Table S1). The percentage of phenotypic variation (coefficient of determination, *R*^2^) of an individual trait explained ranged from 11.75 to 31.32% (Table S1). Under deficit irrigation, 61 markers had significant associations with the studied traits, at the 0.01 probability level (Table S1). The percentage of phenotypic variation (*R*^2^) of a trait explained varied from 9.89 to 26.28% (Table S1). However, in the MLM model, kinship or relatedness matrix was considered as a factor, and the number of significant markers decreased as compared to GLM model. So that, under normal irrigation 57 markers and under deficit irrigation 54 markers indicated significant associations at the 0.01 probability level (Table 4). In this model, the percentage of phenotypic variation, under normal irrigation ranged from 7.71 to 20.89% and under deficit irrigation varied from 6.76 to 17.61% (Table 4). Moreover, association analysis was also done for drought tolerance and susceptibility indices. Results revealed that 19 and 20 markers showed significant associations with the calculated indices based on GLM and MLM model, respectively (Tables 5 and S2).

**Table 4:** Association of SRAP markers with phenological, morphological, and agronomic traits of smooth bromegrass genotypes under normal and water deficit conditions based on mixed linear model (MLM)

Traits	Normal irrigation			Deficit irrigation		
	Marker	*P* value	R^2^ (%)	Marker	*P* value	R^2^ (%)
DPE	Me1/Em4–13^a^	0.0054	10.08	Me4/Em1–5^a^	0.0047	9.52
	Me3/Em1–13	0.0058	9.94	Me4/Em2–11	0.0049	9.46
	Me2/Em1–14	0.0059	9.88	Me2/Em4–7	0.0062	9.01
	Me2/Em2–13	0.0067	9.61	Me1/Em4–13^a^	0.0080	8.52
	Me4/Em1–5^a^	0.0070	9.54			
	Me1/Em3–10	0.0098	8.82			
DA	Me1/Em6–7^a^	0.0012	11.20	Me1/Em6–7^a^	0.0001	12.79
	Me2/Em1–14^a^	0.0013	10.99	Me2/Em1–14^a^	0.0004	11.33
	Me1/Em4–13	0.0017	10.56	Me2/Em1–12	0.0011	9.87
	Me2/Em2–13^a^	0.0032	9.53	Me4/Em6–22^a^	0.0017	9.23
	Me4/Em6–22^a^	0.0051	8.70	Me2/Em5–21^a^	0.0043	7.82
	Me3/Em1–13	0.0069	8.15	Me5/Em3–3	0.0085	6.78
	Me2/Em5–21^a^	0.0088	7.71	Me2/Em2–13^a^	0.0086	6.76
PH	Me1/Em6–7^a^	0.0008	20.89	Me1/Em6–7^a^	0.0016	18.83
	Me2/Em1–14	0.0013	19.40	Me2/Em5–21^a^	0.0024	17.61
	Me2/Em5–21^a^	0.0014	19.20	Me5/Em5–15	0.0046	15.68
	Me5/Em3–10	0.0046	15.59	Me1/Em2–2	0.0067	14.51
	Me5/Em6–1	0.0060	14.79	Me4/Em2–11	0.0070	14.35
	Me4/Em3–14	0.0062	14.67			
	Me2/Em4–2	0.0072	14.21			
	Me2/Em5–22	0.0084	13.75			
FLL	Me2/Em5–21	0.0011	16.46	Me1/Em6–7^a^	0.0027	14.97
	Me1/Em6–7^a^	0.0015	15.62	Me2/Em5–8	0.0029	14.86
	Me5/Em6–11	0.0025	14.46	Me3/Em1–6	0.0047	13.53
	Me5/Em3–3	0.0045	12.96	Me2/Em1–20	0.0061	12.83
	Me2/Em1–14	0.0054	12.47	Me2/Em2–4	0.0063	12.74
	Me1/Em6–16	0.0075	11.62	Me2/Em4–23	0.0077	12.19
				Me5/Em1–21	0.0078	12.17
				Me4/Em3–13	0.0087	11.85
FLW	Me4/Em4–14^a^	0.0011	14.76	Me1/Em2–21^a^	0.0011	14.70
	Me4/Em6–2^a^	0.0012	14.51	Me4/Em6–2^a^	0.0023	13.12
	Me5/Em2–1^a^	0.0014	14.09	Me4/Em4–14^a^	0.0029	12.59
	Me1/Em2–21^a^	0.0042	11.73	Me5/Em2–1^a^	0.0047	11.52
	Me1/Em6–7	0.0042	11.71	Me4/Em1–14	0.0068	10.65
	Me5/Em5–7	0.0072	10.48	Me1/Em1–13^a^	0.0093	9.93
	Me1/Em1–13^a^	0.0079	10.27			
PL	Me2/Em3–5	0.0049	13.64	Me5/Em2–13	0.0015	15.49
	Me4/Em1–9	0.0084	12.13	Me2/Em2–18	0.0043	12.93
				Me2/Em2–12	0.0075	11.53
				Me1/Em1–6	0.0088	11.12
NS	Me2/Em2–16	0.0033	16.43	Me5/Em4–10	0.0027	17.56
	Me2/Em6–18	0.0039	15.98	Me4/Em2–24	0.0030	17.26
	Me5/Em4–7^a^	0.0041	15.83	Me5/Em4–7^a^	0.0034	16.92
	Me5/Em4–11	0.0049	15.23	Me5/Em2–20	0.0048	15.79
	Me2/Em6–10	0.0084	13.57	Me4/Em6–20	0.0096	13.59
	Me4/Em3–11	0.0094	13.25			
DMY	Me1/Em3–15	0.0030	14.42	Me1/Em5–11^a^	0.0028	14.14
	Me4/Em2–12	0.0061	12.61	Me1/Em5–23	0.0033	13.75
	Me4/Em1–17	0.0081	11.84	Me2/Em4–16	0.0098	10.93
	Me1/Em5–11^a^	0.0094	11.43			
CD	Me4/Em2–12	0.0004	20.20	Me5/Em4–22^a^	0.0040	13.52
	Me5/Em4–11	0.0049	13.87	Me1/Em5–11	0.0050	12.95
	Me5/Em4–22^a^	0.0095	12.00	Me2/Em4–16	0.0095	11.24
WGV	Me1/Em5–11^a^	0.0011	19.30	Me2/Em4–7	0.0024	17.21
	Me5/Em4–21	0.0015	18.42	Me1/Em5–11^a^	0.0032	16.35
	Me5/Em3–5	0.0028	16.69	Me5/Em5–18	0.0040	15.70
	Me5/Em2–20^a^	0.0036	15.98	Me2/Em6–16^a^	0.0046	15.32
	Me1/Em5–24	0.0045	15.31	Me5/Em2–20^a^	0.0046	15.30
	Me5/Em1–4	0.0047	15.14	Me1/Em5–3	0.0048	15.16
	Me2/Em6–16^a^	0.0094	13.04	Me1/Em2–19	0.0058	14.59
	Me2/Em2–1	0.0099	12.86	Me2/Em5–5	0.0074	13.84
				Me1/Em3–15	0.0089	13.27

*CD* Crown diameter, *DA* Days to anthesis, *DMY* Dry matter yield, *DPE* Days to panicle emergence, *FLL* Flag leaf length, *FLW* Flag leaf width, *NS* Number of stems per plant, *PH* Plant height, *PL* Panicle length, *WGV* Winter growth vigor

^a^ Stable markers under normal and water deficit conditions

**Table 5:** Association of SRAP markers with drought tolerance and susceptibility indices of smooth bromegrass genotypes based on mixed linear model (MLM)

Indices	Marker	*P* value	R^2^ (%)
TOL	Me5/Em5–16	0.0009	19.96
	Me1/Em2–1	0.0013	19.00
	Me2/Em6–8	0.0013	18.94
	Me2/Em4–16	0.0032	16.37
	Me4/Em2–12	0.0049	15.10
	Me1/Em2–6	0.0062	14.38
	Me5/Em6–3	0.0064	14.27
	Me2/Em6–9	0.0098	12.98
MP	Me1/Em5–11	0.0024	14.67
	Me1/Em3–15	0.0044	13.17
GMP	Me1/Em5–11	0.0022	15.04
	Me1/Em3–15	0.0063	12.28
	Me5/Em4–7	0.0088	11.41
DSI	Me5/Em5–16	0.0016	18.11
	Me5/Em5–9	0.0062	14.16
	Me1/Em2–6	0.0064	14.05
	Me5/Em3–10	0.0064	14.03
STI	Me1/Em5–11	0.0015	17.10
	Me5/Em4–7	0.0055	13.61
	Me1/Em3–15	0.0097	11.98

*DSI* Drought susceptibility index, *GMP* Geometric mean productivity, *MP* Mean productivity, *STI* Stress tolerance index, *TOL* Tolerance index

Based on the results of GLM and MLM models, some markers were associated with more than one trait at the same time. For example, under normal and deficit irrigation regimes, marker Me1/Em6–7 showed simultaneously significant associations with DA, PH, FLL, and FLW, based on both GLM and MLM models. Marker Me2/Em5–21 had concurrently significant associations with DA, PH, and FLL, under both irrigation regimes and in both GLM and MLM models (Tables 4 and S1). In addition, under normal irrigation, marker Me2/Em1–14 showed significant associations with DPE, DA, PH, FLL, and FLW, based on GLM model; and showed significant associations with the traits of DPE, DA, PH, and FLL based on MLM model. However, under deficit irrigation this marker had significant associations with DA and PH based on both models (Tables 4 and S1). Based on MLM model, marker Me1/Em5–11 showed associations with DMY and WGV under both normal and deficit irrigation regimes (Table 4). Moreover, markers Me1/Em5–11 and Me1/Em3–15 showed significant associations with MP, GMP, and STI, based on both GLM and MLM models (Tables 5 and S2).

To assess stable associations, association analysis was conducted in each irrigation regime, separately. In total, 30 and 21 MTAs showed adequately stable expression across irrigation regimes, based on GLM and MLM models, respectively. For instance, in GLM model, markers Me1/Em6–7, Me2/Em1–14, Me2/Em2–13, Me4/Em6–22, Me2/Em5–21, Me2/Em3–19, Me2/Em1–12, and Me1/Em6–16 showed significant and stable associations in both irrigation regimes with DA. Similarly, markers Me1/Em5–11, Me1/Em5–24, Me2/Em6–16, Me5/Em2–20, and Me1/Em2–19 displayed significant and constant associations in both irrigation regimes with WGV (Table S1). In MLM model, markers Me1/Em6–7, Me2/Em1–14, Me2/Em2–13, Me4/Em6–22, and Me2/Em5–21 had significant and stable associations in both irrigation regimes with DA. Also, markers Me4/Em4–14, Me4/Em6–2, Me5/Em2–1, Me1/Em2–21, and Me1/Em1–13 were constantly associated with FLW in both moisture conditions. In the same way, marker Me1/Em5–11 was associated with DMY (Table 4).

## Discussion

Significant genetic variations among genotypes in terms of all of the evaluated traits demonstrate the difference in genes controlling yield, its components, and drought-tolerance. Moreover, the non-static performance of genotypes in two irrigation regimes emphasizes the importance of marker-trait association analysis in the two moisture environments, separately.

Most of the evaluated traits were significantly affected by water deficit due to decreased water potential of the soil and decline in net assimilation and photosynthesis of leaves [37, 38]. Similar results were reported in other researches [39, 40]. Wide genetic variation observed for all of the evaluated traits is promising genetic progress for these genotypes. Moreover, higher estimates for PCV and GCV under the deficit irrigation regime compared with normal irrigation indicate that water deficit have increased genetic variation for most of the studied traits and therefore, selection under deficit irrigation would be more effective. Our findings in this case are consistent with the reports of Abtahi et al. [41] and Saeidnia et al. [42]. However, some researchers stated that the genetic advance through selection is higher under normal irrigation [39, 43]. Moderate to high heritability estimates observed for all of the evaluated traits indicates that the improvement of these traits would be possible through selection and also emphasizes that detecting of marker–trait associations is possible for these traits [44].

The high percentage of polymorphism indicated that SRAP markers used in the present research could be used as powerful tools for discriminating of smooth bromegrass genotypes. Results of this study also revealed that the primer combination Me4/Em2 with the highest polymorphism percentage and high values of PIC, MI, and RP indices is informative and powerful enough for identification and discrimination of smooth bromegrass genotypes.

Population structure analysis identified three groups of genotypes in the studied panel of smooth bromegrass. As expected, structure analysis was able to separate genotypes based on their origin. Based on the results of association analysis of different traits under normal and deficit irrigations, the number of significant MTAs was lower in the MLM than GLM model. SRAP markers identified based on the results of MLM model can be considered as the most interesting candidates for future studies using MAS. It is stated that the combination of Q and K matrices strongly reduces the coefficient of determination and likely provides the best correction for population structure [45].

Marker-trait associations were mostly different in normal and deficit irrigation regimes. The results showed that a greater number of genes were probably involved in controlling traits at deficit irrigation regime than normal one. The percentage of variation which is explained by identified associations was low (7.71–20.89% under normal irrigation and 6.76–17.61% under water-deficit irrigation). This low *R*^2^ value for each trait may be attributed to the role of many minor genes controlling the trait, outcrossing nature of smooth bromegrass, markers exhibiting minor quantitative effect, rare alleles, and complex allelic interactions [46, 47]. These results are in agreement with the findings of Lou et al. [32] and Sun et al. [33] in tall fescue.

Based on the results of GLM and MLM models, some markers had simultaneously significant associations with more than one trait. These markers may be effectively used to improve several traits, concurrently [33, 34]. Multi-association between different traits could be attributed to the co-expression mediated by expression of quantitative trait loci or e-QTLs [48]. For instance, marker Me1/Em6–7 showed simultaneously significant associations with DA, PH, FLL, and FLW, under both irrigation regimes and based on both GLM and MLM models. Similarly, marker Me2/Em5–21 concurrently showed significant associations with DA, PH, and FLL. In addition, under normal irrigation, marker Me2/Em1–14 showed significant associations with DPE, DA, PH, FLL, and FLW, based on GLM model; and also showed significant associations with DPE, DA, PH, and FLL based on MLM model. At deficit irrigation, this marker had significant associations with DA and PH based on both models. These simultaneous associations of markers with multiple traits may be attributed to pleiotropic effects or to several tightly linked genes that affect multiple traits [33, 49].

Determination of the genetic basis of drought tolerance requires correlating the occurrence of molecular markers with phenotypic scores for prediction of DNA genomic regions which involves effective factors on the response of plants [50]. Marker–trait association analysis identified 19 and 20 loci related with drought tolerance and susceptibility indices based on GLM and MLM models, respectively. Among these, SRAP markers Me1/Em5–11 and Me1/Em3–15 showed significant associations with MP, GMP, and STI, based on both GLM and MLM models. Moreover, in both models, markers Me5/Em5–9 and Me5/Em3–10 showed a significant association with DSI. If the effectiveness of these regions in the genetic control of drought tolerance is confirmed, these markers could be potentially used for the improvement of drought tolerance in smooth bromegrass.

Most of the MTAs were different under normal and deficit irrigations, indicating that the environmental factors have affected these associations [51]. These results showed that different genes may be effective on the same trait in different environments [52] or there might be a change in the expression level of the same gene between the two environments [48]. In the present study, 21 markers showed stable associations with different traits under both irrigation regimes. Diapari et al. [53] stated that associated markers which were detected in two or more different environments are more reliable than those present in only one environment.

## Conclusion

In conclusion, the efficiency of association analysis approach as a powerful tool for identifying and detecting genes and markers linked to complex traits of agricultural and economic importance was illustrated. Satisfactory levels of polymorphism were observed for the studied traits in the polycrossed population. Three subpopulations were identified in smooth bromegrass genotypes; and 90 significant MTAs were detected using GLM and MLM models, under contrasting water conditions. Among these, three MTAs were identified for drought tolerance. Moreover, it was demonstrated that SRAP markers can be used in the future breeding programs to enhance drought tolerance of smooth bromegrass. Some SRAP markers were associated with the key agronomic traits of this species. Environmental specificity of MTAs shows that genotype × environment interactions are effective on association analysis; nevertheless, 30 and 21 MTAs showed significantly stable expression across two irrigation regimes based on GLM and MLM models, respectively. The markers identified in the present study are useful genomic resources for MAS in the future breeding programs of smooth bromegrass.

## Methods

### Plant materials and field experiment

A replicated nursery of smooth bromegrass (containing 1000 samples) was established at Isfahan University of Technology Research Farm in 2006. Some of these plant materials were provided by the Hungarian Institute for Agrobotany (HIFA), Tapioszele, Hungary. The Iranian ecotypes were natural ecotypes collected from wide geographical areas of Iran by a team which are specialist in the field of grass species. These samples were evaluated at first in the research center of Fozveh, Isfahan, Iran. After formal identification and verification of them, these ecotypes were used for research projects of Isfahan University of Technology (IUT).

Genotype panel used for the present study consisted of 216 clones randomly selected from a large nursery, comprised of 1800 single spaced-plant polycrossed progenies resulting from 36 parental ecotypes of smooth bromegrass (*Bromus inermis*). These 36 genotypes were randomly selected from polycross progenies of a set of 25 parental genotypes (Table 6). The parental genotypes were randomly chosen from the above mentioned nursery. For the present study, 216 clones were propagated in a greenhouse during the winter of 2012 and then were space-planted (50-cm grid) in the field according to a RCBD with six replications. Genotypes were evaluated under two levels of irrigation including a normal and a water deficit condition for 2014–2015. Under normal and water deficit conditions, irrigation was done when 50 and 90% of the total available soil water was exhausted from the root zone, respectively [54].

**Table 6:** Information on the genetic materials used in this study

Genotype	Population code	Origin
1	2000/50	Iran, Isfahan- Fozve
2	2000/50	Iran, Isfahan- Fozve
3	2000/50	Iran, Isfahan- Fozve
4	2000/24	Iran, Isfahan- Fozve
5	2000/T-9	Iran, Hamedan
6	2000/T-9	Iran, Hamedan
7	2000/24	Iran, Isfahan- Fozve
8	2000/T-9	Iran, Hamedan
9	2000/18–2	Iran, Isfahan- Fozve
10	2000/18–2	Iran, Isfahan- Fozve
11	RCAT042134	Hungary
12	RCAT042134	Hungary
13	RCAT064839	Hungary
14	2000/36	Iran, Isfahan- Fozve
15	2000/36	Iran, Isfahan- Fozve
16	2000/36	Iran, Isfahan- Fozve
17	2000/36	Iran, Isfahan- Fozve
18	2000/T-9	Iran, Hamedan
19	2000/18	Iran, Isfahan- Fozve
20	2000/18	Iran, Isfahan- Fozve
21	RCAT041861	Hungary
22	2000/40	Iran, Isfahan- Semirom
23	2000/4	Iran, Isfahan- Fozve
24	2000/30	Iran, Isfahan- Fozve
25	RCAT042133	Hungary
26	RCAT042133	Hungary
27	2000/10	Iran, Kordestan
28	RCAT041861	Hungary
29	2000/10	Iran, Kordestan
30	RCAT064835	Hungary
31	RCAT064835	Hungary
32	RCAT064835	Hungary
33	RCAT064835	Hungary
34	RCAT064837	Hungary
35	RCAT064837	Hungary
36	RCAT064837	Hungary

This experiment was carried out in the field at Research Farm of Isfahan University of Technology, situated in Lavark, Najaf-Abad, Isfahan, Iran (32° 30′ N, 51° 20′ E, 1630 m amsl) during 2 years. This region has a mean annual temperature of 14.6 °C and mean annual precipitation of 141 mm, generally without rain during the summer, making supplemental irrigation necessary for growing crops during this period.

### Phenotyping

During the year that plants were established (2013) no data was recorded. Days to panicle emergence (DPE), days to anthesis (DA), plant height (PH), and winter growth vigor (WGV) were measured as mentioned in our previous studies [39, 55]. Flag leaf length (FLL), flag leaf width (FLW), panicle length (PL), and number of stems per plant (NS) were measured at the pollination stage. After the complete flowering (about early summer), the produced forage of each genotype was harvested by cutting the grass from 5 cm above the ground in late spring, late summer, and late autumn. The harvested materials were then dried at 75 °C for 48 h and weighed to obtain dry matter yield (DMY). The average forage weight (g per plant) from the three cuts was used for analysis. The width of plant basal cover remained after the first harvest was considered as crown diameter (CD).

Five selection indices including tolerance index (TOL) [56], mean productivity (MP) [56], geometric mean productivity (GMP) [57], drought susceptibility index (DSI) [58], and stress tolerance index (STI) [57] were calculated based on the dry matter yield under normal and water deficit irrigations, according to the following formulations:

$$ \mathrm{TOL}={\mathrm{Y}}_{\mathrm{pi}}-{\mathrm{Y}}_{\mathrm{si}} $$

$$ \mathrm{MP}=\left({\mathrm{Y}}_{\mathrm{pi}}+{\mathrm{Y}}_{\mathrm{si}}\right)/2 $$

$$ \mathrm{GMP}={\left({\mathrm{Y}}_{\mathrm{pi}}\times {\mathrm{Y}}_{\mathrm{si}}\right)}^{0.5} $$

$$ \mathrm{DSI}=\left[1-\left({\mathrm{Y}}_{\mathrm{si}}/{\mathrm{Y}}_{\mathrm{pi}}\right)\right]/\left[1-\left({\mathrm{Y}}_{\mathrm{ms}}/{\mathrm{Y}}_{\mathrm{mp}}\right)\right] $$

$$ \mathrm{STI}=\left[\left({\mathrm{Y}}_{\mathrm{pi}}\times {\mathrm{Y}}_{\mathrm{si}}\right)/{\left({\mathrm{Y}}_{\mathrm{mp}}\right)}^2\right] $$

where Y_si_ designates the yield of the ith genotype grown under deficit irrigation, Y_pi_ designates that of the ith genotype grown under normal irrigation, Y_ms_ is the yield mean over all genotypes grown under deficit irrigation, and Y_mp_ is the yield mean over all genotypes grown under normal irrigation.

### Genotyping

Genomic DNA was extracted from young leaf tissues according to the modified method of Murray and Thompson [59]. The quality and concentration of extracted DNA were determined by electrophoresis in 1% agarose gel. Genotyping using SRAP markers was performed following the method of Li and Quiros [9]. Among the SRAP markers available, 30 primer combinations were screened by polymerase chain reaction (PCR). PCR reactions were conducted in volumes of 10 *μ*L, using a BIO-RAD thermocycler. Each PCR reaction was consisted of 1.5 *μ*L of DNA, 1 *μ*L of forward primer, 1 *μ*L of reverse primer, 5 *μ*L of master mix (Amplicon), and 1.5 *μ*L of distilled water. In SRAP analysis, samples were exposed to the following thermic profile: the first five cycles were run at 94 °C for 1 min (denaturing), 35 °C for 1 min (annealing), and 72 °C for 1 min (extension). Then the annealing temperature was raised to 50 °C for another 35 cycles, followed by another extension step of 10 min at 72 °C. Electrophoresis on 12% non-denatured polyacrylamide gels was used for separation of amplified products; and then the products were stained by AgNO3 solution [60].

### Statistical analyses

#### Phenotypic data analysis

Data were tested for normality by Kolmogorov–Smirnov test and homogeneity of variance was tested by Bartlett test. Analysis of variance was performed for the normal and water-deficit irrigations separately, based on the split-plot in time (year) model, using Proc GLM of SAS release 9.4 [61]. Components of variance were calculated for each irrigation regimes, according to the method of Steel and Torrie [62]. Broad-sense heritability (h^2^_b_) was estimated for both irrigation regimes as described by Nguyen and Sleper [63]:

$$ {\mathrm{h}}^2={\upsigma^2}_{\mathrm{g}}/\left({\upsigma^2}_{\mathrm{g}}+{\upsigma^2}_{\mathrm{g}\mathrm{y}}/\mathrm{y}+{\upsigma^2}_{\mathrm{g}\mathrm{r}}/\mathrm{r}+{\upsigma^2}_{\mathrm{e}}/\mathrm{r}\mathrm{y}\right) $$

In which, *σ*^*2*^_*g*_ is the genotype, *σ*^*2*^_*gy*_ is the genotype × year, *σ*^*2*^_*rg*_ is the genotype × rep, and *σ*^*2*^_*e*_ is the residual variance, y is the number of years, and r is the number of replicates. The level of genetic variation was estimated with the calculating of phenotypic coefficient of variation (PCV) and genetic coefficient of variation (GCV) as follows:

$$ \mathrm{PCV}=\left({\upsigma}_{\mathrm{p}}/\upmu \right)\ 100 $$

$$ \mathrm{GCV}=\left({\upsigma}_{\mathrm{g}}/\upmu \right)\ 100 $$

where σ_p_ is the standard deviation of the phenotypic variance, σ_g_ is the standard deviation of the genotypic variance, and μ is the phenotypic mean [64].

#### Molecular data analysis

Polymorphic SRAP markers were scored as binary data with presence (1) or absence (0). For each of the SRAP markers, the following indices were computed. Polymorphism information content (PIC) was determined according to the formula of Roldán-Ruiz et al. [65]:

$$ {\mathrm{PIC}}_{\mathrm{i}}=\Big({2\mathrm{f}}_{\mathrm{i}}\times \left[1-\mathrm{f}\right] $$

where i is the ith primer, f_i_ is the frequency of the amplified allele, and (1-f_i_) is the frequency of the null allele. Resolving power (RP) was estimated as:

$$ \mathrm{RP}=\sum 1-\left[2\times \left(0.5-{\mathrm{f}}_{\mathrm{i}}\right)\right] $$

Marker index (MI) was calculated according to Powell et al. [66]:

$$ {\mathrm{MI}}_{\mathrm{i}}={\mathrm{PIC}}_{\mathrm{i}}\times {\mathrm{N}}_{\mathrm{i}}\times {\mathrm{b}}_{\mathrm{i}} $$

where N_i_ is the total bands for the ith primer, and b_i_ is the percentage polymorphic bands of the ith primer.

#### Population structure and association analysis

Structure analysis and stratification of the studied population into subpopulations with different genetic structures was done based on SRAP marker data in STRUCTURE software version 2.3.4 [25]. This analysis was performed applying an admixture model, a burn-in of 10,000 iterations followed by 100,000 Monte Carlo Markov Chain (MCMC) replicates. The membership of each genotype was run for the range of genetic clusters (K) from K = 2 to K = 10 with five replications for each *K. delta* k approach described by Evanno et al. [67] was used to determine the optimum number of sub-populations, using STRUCTURE HARVESTER [68].

Association analysis was run by both GLM and MLM [26] to calculate *P*-values for marker–trait associations, using TASSEL version 4.2.1 [69]. The phenotypic mean of traits (P-matrix) over 2 years was used to identify significant associations under normal and water deficit irrigations, separately. To correct for population structure in GLM and MLM models, a Q-matrix that was derived from structure analysis (at maximum DK), was applied as a covariate. Moreover, a kinship matrix (K-matrix) was calculated based on the results of marker genotype data using TASSEL version 4.2.1 [69] and was used in MLM. A correction for multiple testing was done with the FDR (false discovery rate) method, using the QVALUE R package [70].

## Supplementary Information

**Additional file 1.**

## Data Availability

The datasets used and/or analyzed during the current study are presented in the additional supporting file. Data also can be accessed in IUT box of Isfahan University of Technology, Isfahan, Iran (https://iutbox.iut.ac.ir/index.php/s/Y3pWLqm8QF4SYbr).

## Abbreviations

CD: Crown diameter
DA: Days to anthesis
DMY: Dry matter yield
DPE: Days to panicle emergence
DSI: Drought susceptibility index
FLL: Flag leaf length
FLW: Flag leaf width
GCV: Genetic coefficient of variation
GLM: General linear model
GMP: Geometric mean productivity
GWAS: Genome wide association study
MAS: Marker assisted selection
MLM: Mixed linear model
MP: Mean productivity
MTA: Marker-trait association
NS: Number of stems per plant
PCV: Phenotypic coefficient of variation
PH: Plant height
PL: Panicle length
QTL: Quantitative trait loci
SRAP: Sequence related amplified polymorphism
STI: Stress tolerance index
TOL: Tolerance index
WGV: Winter growth vigor
