# Supplementary material for: Marker-trait association analysis for drought tolerance in smooth bromegrass
Source: BMC Plant Biol. 2021 Feb 25;21:116. doi: 10.1186/s12870-021-02891-0 (PMC7908751; doi:10.1186/s12870-021-02891-0)
Supplement: Supplementary file 1 — Additional file 1. [file 12870_2021_2891_MOESM1_ESM.doc]

| **Table S1** Association of SRAP markers with phenological, morphological, and agronomic traits of smooth bromegrass genotypes under normal and water deficit conditions based on general linear model (GLM) | | | | | | | |
| --- | --- | --- | --- | --- | --- | --- | --- |
| Traits | Normal irrigation | | |  | Deficit irrigation | | |
|  | Marker | P value | R^2^ (%) |  | Marker | P value | R^2^ (%) |
| DPE | Me2/Em1-14* | 0.0032 | 16.14 |  | Me1/Em4-13* | 0.0060 | 13.01 |
|  | Me1/Em4-13* | 0.0034 | 16.01 |  | Me4/Em2-11 | 0.0060 | 13.02 |
|  | Me2/Em2-13 | 0.0043 | 15.34 |  | Me2/Em4-7 | 0.0067 | 12.71 |
|  | Me3/Em1-13 | 0.0069 | 13.86 |  | Me4/Em1-5* | 0.0069 | 12.62 |
|  | Me1/Em3-10 | 0.0086 | 13.22 |  | Me4/Em5-18* | 0.0086 | 12.01 |
|  | Me4/Em5-18* | 0.0086 | 13.20 |  | Me2/Em1-14* | 0.0099 | 11.62 |
|  | Me4/Em1-5* | 0.0087 | 13.19 |  |  |  |  |
|  |  |  |  |  |  |  |  |
| DA | Me1/Em6-7* | 0.0005 | 19.01 |  | Me1/Em6-7* | 0.00005 | 20.60 |
|  | Me2/Em1-14* | 0.0005 | 18.92 |  | Me2/Em1-14* | 0.0002 | 18.10 |
|  | Me1/Em4-13 | 0.0012 | 17.00 |  | Me2/Em1-12* | 0.0005 | 16.16 |
|  | Me2/Em2-13* | 0.0015 | 16.40 |  | Me4/Em6-22* | 0.0012 | 14.50 |
|  | Me4/Em6-22* | 0.0033 | 14.33 |  | Me2/Em5-21* | 0.0024 | 12.96 |
|  | Me2/Em5-21* | 0.0045 | 13.48 |  | Me5/Em3-3 | 0.0048 | 11.40 |
|  | Me2/Em3-19* | 0.0061 | 12.69 |  | Me2/Em2-13* | 0.0054 | 11.14 |
|  | Me2/Em1-12* | 0.0067 | 12.42 |  | Me1/Em6-16* | 0.0056 | 11.06 |
|  | Me5/Em6-9 | 0.0068 | 12.37 |  | Me5/Em2-5 | 0.0059 | 10.96 |
|  | Me3/Em1-13 | 0.0076 | 12.09 |  | Me5/Em3-17 | 0.0069 | 10.58 |
|  | Me1/Em6-16* | 0.0086 | 11.75 |  | Me4/Em1-19 | 0.0080 | 10.24 |
|  |  |  |  |  | Me1/Em5-1 | 0.0087 | 10.05 |
|  |  |  |  |  | Me2/Em3-19* | 0.0089 | 9.98 |
|  |  |  |  |  | Me2/Em2-7 | 0.0093 | 9.89 |
|  |  |  |  |  |  |  |  |
| PH | Me1/Em6-7* | 0.0006 | 31.32 |  | Me1/Em6-7* | 0.0016 | 26.28 |
|  | Me2/Em1-14 | 0.0013 | 28.13 |  | Me2/Em5-21* | 0.0027 | 24.11 |
|  | Me2/Em5-21* | 0.0015 | 27.40 |  | Me5/Em5-15 | 0.0063 | 20.53 |
|  | Me5/Em3-10 | 0.0033 | 23.99 |  | Me4/Em2-11 | 0.0068 | 20.20 |
|  | Me2/Em5-22 | 0.0057 | 21.54 |  | Me3/Em1-2 | 0.0087 | 19.11 |
|  | Me4/Em3-14 | 0.0057 | 21.52 |  | Me1/Em2-2 | 0.0091 | 18.89 |
|  | Me5/Em6-1 | 0.0061 | 21.23 |  |  |  |  |
|  | Me2/Em4-2 | 0.0089 | 19.51 |  |  |  |  |
|  |  |  |  |  |  |  |  |
| FLL | Me2/Em5-21 | 0.0006 | 26.96 |  | Me1/Em6-7* | 0.0017 | 23.76 |
|  | Me1/Em6-7* | 0.0006 | 26.80 |  | Me2/Em5-8* | 0.0029 | 21.86 |
|  | Me2/Em1-14 | 0.0018 | 23.10 |  | Me2/Em2-4* | 0.0037 | 20.90 |
|  | Me5/Em6-11 | 0.0018 | 23.01 |  | Me3/Em1-6 | 0.0045 | 20.08 |
|  | Me5/Em3-3 | 0.0028 | 21.43 |  | Me5/Em1-21 | 0.0057 | 19.15 |
|  | Me1/Em6-16* | 0.0030 | 21.25 |  | Me4/Em3-13 | 0.0062 | 18.78 |
|  | Me5/Em6-9 | 0.0065 | 18.19 |  | Me1/Em6-16* | 0.0065 | 18.59 |
|  | Me2/Em2-4* | 0.0079 | 17.43 |  | Me2/Em1-20 | 0.0066 | 18.54 |
|  | Me2/Em5-8* | 0.0085 | 17.14 |  | Me5/Em3-8 | 0.0073 | 18.15 |
|  | Me5/Em3-10 | 0.0095 | 16.68 |  |  |  |  |

| **Table S1** (continued) | | | | | | | |
| --- | --- | --- | --- | --- | --- | --- | --- |
| Traits | Normal irrigation | | |  | Deficit irrigation | | |
|  | Marker | P value | R^2^ (%) |  | Marker | P value | R^2^ (%) |
| FLW | Me4/Em4-14* | 0.0007 | 22.36 |  | Me1/Em2-21* | 0.0011 | 21.12 |
|  | Me4/Em6-2* | 0.0008 | 22.02 |  | Me4/Em4-14* | 0.0020 | 19.20 |
|  | Me5/Em2-1* | 0.0011 | 21.21 |  | Me4/Em6-2* | 0.0028 | 18.21 |
|  | Me1/Em6-7 | 0.0029 | 18.12 |  | Me5/Em2-1* | 0.0054 | 16.05 |
|  | Me1/Em2-21* | 0.0039 | 17.17 |  | Me1/Em1-13* | 0.0069 | 15.23 |
|  | Me5/Em5-7 | 0.0063 | 15.61 |  |  |  |  |
|  | Me1/Em1-13* | 0.0064 | 15.55 |  |  |  |  |
|  | Me2/Em1-14 | 0.0100 | 14.05 |  |  |  |  |
|  |  |  |  |  |  |  |  |
| PL | Me2/Em3-5 | 0.0060 | 18.15 |  | Me5/Em2-13 | 0.0016 | 21.37 |
|  | Me4/Em1-9 | 0.0079 | 17.11 |  | Me2/Em2-18 | 0.0065 | 16.57 |
|  |  |  |  |  |  |  |  |
| NS | Me2/Em6-18 | 0.0020 | 25.68 |  | Me4/Em2-24 | 0.0026 | 24.88 |
|  | Me2/Em2-16 | 0.0030 | 24.00 |  | Me5/Em4-10 | 0.0026 | 24.88 |
|  | Me2/Em6-10 | 0.0051 | 21.76 |  | Me5/Em4-7* | 0.0060 | 21.18 |
|  | Me5/Em4-11 | 0.0065 | 20.69 |  | Me5/Em2-20 | 0.0071 | 20.43 |
|  | Me5/Em4-7* | 0.0072 | 20.22 |  |  |  |  |
|  |  |  |  |  |  |  |  |
| DMY | Me1/Em3-15 | 0.0030 | 20.92 |  | Me1/Em5-23 | 0.0024 | 20.67 |
|  | Me4/Em1-17 | 0.0057 | 18.42 |  | Me1/Em5-11 | 0.0043 | 18.60 |
|  | Me4/Em2-12 | 0.0069 | 17.71 |  |  |  |  |
|  |  |  |  |  |  |  |  |
| CD | Me4/Em2-12 | 0.0004 | 28.79 |  | Me5/Em4-22* | 0.0026 | 20.79 |
|  | Me5/Em4-11 | 0.0040 | 20.48 |  | Me1/Em5-11 | 0.0098 | 15.89 |
|  | Me5/Em4-22* | 0.0069 | 18.32 |  | Me2/Em5-18 | 0.0098 | 15.88 |
|  | Me2/Em5-12 | 0.0099 | 16.87 |  |  |  |  |
|  |  |  |  |  |  |  |  |
| WGV | Me5/Em4-21 | 0.0013 | 28.07 |  | Me5/Em5-18 | 0.0033 | 23.80 |
|  | Me1/Em5-11* | 0.0014 | 27.62 |  | Me2/Em6-16* | 0.0034 | 23.65 |
|  | Me5/Em3-5 | 0.0025 | 25.06 |  | Me1/Em5-11* | 0.0036 | 23.48 |
|  | Me5/Em1-4 | 0.0032 | 24.02 |  | Me2/Em4-7 | 0.0039 | 23.06 |
|  | Me1/Em5-24* | 0.0035 | 23.64 |  | Me1/Em2-19* | 0.0058 | 21.28 |
|  | Me2/Em6-16* | 0.0056 | 21.60 |  | Me5/Em2-20* | 0.0060 | 21.13 |
|  | Me5/Em2-20* | 0.0061 | 21.22 |  | Me2/Em5-5 | 0.0061 | 21.06 |
|  | Me2/Em2-1 | 0.0073 | 20.35 |  | Me1/Em5-3 | 0.0062 | 21.00 |
|  | Me4/Em2-17 | 0.0075 | 20.24 |  | Me1/Em5-24* | 0.0063 | 20.97 |
|  | Me1/Em2-19* | 0.0097 | 19.07 |  | Me1/Em3-15 | 0.0070 | 20.48 |
| * Stable markers under normal and water deficit conditions  CD, Crown diameter; DA, Days to anthesis; DMY, Dry matter yield; DPE, Days to panicle emergence; FLL, Flag leaf length; FLW, Flag leaf width; NS, Number of stems per plant; PH, Plant height; PL, Panicle length; WGV, Winter growth vigor | | | | | | | |

| **Table S2** Association of SRAP markers with drought tolerance and susceptibility indices of smooth bromegrass genotypes based on general linear model (GLM) | | | |
| --- | --- | --- | --- |
| Indices | Marker | P value | R^2^ (%) |
| TOL | Me5/Em5-16 | 0.0009 | 29.33 |
|  | Me2/Em6-8 | 0.0010 | 28.82 |
|  | Me1/Em2-1 | 0.0012 | 27.94 |
|  | Me2/Em4-16 | 0.0023 | 25.24 |
|  | Me1/Em2-6 | 0.0055 | 21.49 |
|  | Me5/Em6-3 | 0.0062 | 20.98 |
|  | Me4/Em2-12 | 0.0083 | 19.68 |
|  | Me2/Em6-9 | 0.0088 | 19.38 |
|  |  |  |  |
| MP | Me1/Em5-11 | 0.0040 | 19.18 |
|  | Me1/Em3-15 | 0.0042 | 19.01 |
|  |  |  |  |
| GMP | Me1/Em5-11 | 0.0035 | 19.72 |
|  | Me1/Em3-15 | 0.0064 | 17.49 |
|  |  |  |  |
|  |  |  |  |
| DSI | Me5/Em5-16 | 0.0013 | 27.29 |
|  | Me5/Em5-9 | 0.0038 | 22.81 |
|  | Me5/Em3-10 | 0.0071 | 20.06 |
|  | Me1/Em2-6 | 0.0089 | 19.06 |
|  |  |  |  |
| STI | Me1/Em5-11 | 0.0028 | 22.10 |
|  | Me5/Em4-7 | 0.0088 | 17.54 |
|  | Me1/Em3-15 | 0.0098 | 17.12 |
|  |  |  |  |
| DSI, Drought susceptibility index; GMP, Geometric mean productivity; MP, Mean productivity; STI, Stress tolerance index; TOL, Tolerance index | | | |
